# Uncovering tomato candidate genes associated with drought tolerance using *Solanum pennellii* introgression lines

**DOI:** 10.1371/journal.pone.0287178

**Published:** 2023-06-15

**Authors:** Herika Paula Pessoa, Françoise Dalprá Dariva, Mariane Gonçalves Ferreira Copati, Ramon Gonçalves de Paula, Felipe de Oliveira Dias, Carlos Nick Gomes

**Affiliations:** 1 Department of Agronomy, Universidade Federal de Viçosa, Viçosa, Minas Gerais, Brazil; 2 Departament of General Biology, Universidade Federal de Viçosa, Viçosa, Minas Gerais, Brazil; Central Research Institute for Dryland Agriculture, INDIA

## Abstract

Tomato plants are sensitive to drought stress throughout their growth cycle. To be considered drought-tolerant, a cultivar should display tolerance at all developmental stages. This study aimed to evaluate whether *Solanum pennellii* introgression lines (ILs) previously selected as drought-tolerant during germination/seedling growth maintained this tolerance in the vegetative/reproductive stage. We then investigated these ILs to uncover candidate genes. The plants were subjected to two different environmental conditions: well-watered and drought-stressed (water withheld for ≤ 20 d after flowering). Phenotyping for morphological, physiological, fruit quality, and yield-related traits was performed, and the data was analyzed using a mixed-model approach. Using a multi-trait index that relies on factor analysis and genotype-ideotype distance (FAI-BLUP index), the genotypes were ordered based on how far they were from the drought-tolerant ideotype. Afterward, the tomato IL population map furnished by the SOL Genomics Network was utilized to identify introgressed segments of significance for the identification of candidate genes. Significant genotypic differences were found in the yield, water content, mean weight, length, and width of the fruit, the percentage of fruits displaying blossom-end rot, and titratable acidity. The drought-tolerance ideotype was built considering the maximum values for the fruit water content, number of fruits, mean fruit weight, and yield, minimum values for blossom-end rot, and mean values for titratable acidity. IL 1-4-18, IL 7-4-1, IL 7–1, IL 7-5-5, and IL 1–2 were ranked above M-82 and therefore considered drought-tolerant during the vegetative/reproductive stage. IL 1-4-18 and IL1-2 sustained drought tolerance displayed during germination/seedling growth into the vegetative/reproductive stage. The following candidate genes associated with drought tolerance were identified: *AHG2*, *At1g55840*, *PRXIIF*, *SAP5*, *REF4-RELATED 1*, *PRXQ*, *CFS1*, *LCD*, *CCD1*, and *SCS*. Because they are already associated with genetic markers, they can be transferred to elite tomato cultivars through marker-assisted technology after validation.

## Introduction

*Solanum lycopersicum* L., the domesticated tomato, is a popular vegetable crop produced and consumed worldwide and is one of the world’s most economically important vegetables [1]. Tomato plants are sensitive to drought stress from seed germination to harvest [2] demanding a high water supply throughout the growing cycle [3, 4]. As in other crops, even short periods of drought might cause tomato growers qualitative and quantitative losses in fruit production [5, 6]. Overall, water shortages can adversely affect tomato plant growth as well as physiological traits, and yield [5–7]. Therefore, anticipating farmer needs, the development of tomatoes possessing drought tolerance has gained attention in tomato breeding programs [8–10].

One of the challenges delaying the release of drought-tolerant cultivars is that this trait seems to be a stage-specific phenomenon [11]. Nevertheless, an ideal drought-tolerant genotype must display drought resistance at all developmental stages of plant growth [12]. Therefore, specific stages throughout the ontogeny of the plant must be evaluated separately to assess drought tolerance and to identify its genetic components [13]. Also, drought tolerance seems to be controlled by many genes with different effects and is highly influenced by environmental variation [14, 15]. Since environmental conditions such as rainfall, temperature, salinity, and nutrient availability cannot be fully controlled [16], identifying candidate genes associated with drought tolerance, and implementing marker assisted selection can increase the precision of the selection of promising drought-tolerant genotypes.

In terms of drought tolerance, the primary genetic variation sources in tomatoes are the wild green-fruited relatives *Solanum chilense* L. and *Solanum pennellii* Corr [13, 17, 18]. However, several crossing barriers with cultivated tomatoes make it impractical to use *S*. *chilense* in breeding programs [19]. Unlike *S*. *chilense*, *S*. *pennellii* is a crossable wild relative, but direct crosses with cultivated tomatoes can result in the undesired linkage drag of traits associated with fruit quality and yield [12].

A viable way to overcome this problem is through the exploitation of the *S*. *pennellii* genome available with a background of domesticated tomatoes in the collection of introgression lines (ILs) [20]. In this collection, defined genomic segments of *S*. *pennellii* have replaced homologous regions in the commercial cultivar M-82, providing complete coverage of the wild species genome. ILs are a valuable genetic resource for studying drought tolerance and identifying the genomic regions associated with it because any phenotypic differences found between an IL and the recurrent parent M-82 when subjected to drought conditions can be solely attributed to the introgressed chromosomal segment of the drought-tolerant *S*. *pennellii* [21, 22].

To aid and accelerate the development of drought-tolerant cultivars, we evaluated *S*. *pennellii* ILs that were previously selected based on their level of drought tolerance during their germination and seedling stages [23]. ILs were exposed to drought by withholding water during the vegetative and reproductive stages and were ranked according to their drought tolerance. Our main goal was to provide information on whether the previously selected drought-resistant genotypes can sustain their tolerance when drought stress is applied in the vegetative and reproductive stages and should, therefore, be incorporated in the next phases of tomato breeding programs. Moreover, the introgressed *S*. *pennellii* fragments of the genotypes selected as drought-tolerant were analyzed and candidate genes associated with this trait in the vegetative and reproductive stages were identified.

## Materials and methods

### Plant materials and greenhouse experiments

To evaluate the response of genotypes to drought stress two experiments with similar conditions, except for irrigation (environmental condition), were conducted simultaneously. On environmental condition 1, plants were kept in non-stressed conditions throughout the growing season, with the soil water content maintained at field capacity. On environmental condition 2 plants were challenged with drought by withholding water for up to 20 d after flowering [24].

Plant materials were chosen based on a previous study [23] that evaluated drought tolerance of ILs from the *S*. *pennellii* population previously in developed [20] during germination and seedling stages. The five ILs previously ranked as the most drought-tolerant (IL 1-4-18, IL 2–3, IL 1–2, IL 9–2, and IL 10–1) and the five ranked as the most drought-sensitive (IL 8–3, IL 7-4-1, IL 7-5-5, IL 9–3, and IL 7–1) together with one of their parents, the commercial variety M-82, were evaluated in this study (Table 1).

**Table 1:** List of genotypes evaluated.

Drought tolerance at germination and seedling stages*	Genotype	Introgression line
Drought tolerant	1	IL 1-4-18
	2	IL 2–3
	3	IL 1–2
	4	IL 9–2
	5	IL 10–1
Drought sensitive	6	IL 8–3
	7	IL 7-4-1
	8	IL 7-5-5
	9	IL 9–3
	10	IL 7–1
**Parent**	**11**	**cv. M-82**

*Drought tolerance and/or sensitivity were determined in a previous study [23].

The greenhouse experiment was conducted in the Research and Extension Farm Unit *Horta Velha* at the Universidade Federal de Viçosa, Viçosa, MG, Brazil (20° 45′ 14″ S; 42° 52′ 53″ W; 648.74 m altitude) from July to December 2019. Temperature (T, °C) and relative air humidity (RH, %) inside the greenhouse were monitored daily using the weather station E4000 (Irriplus ^®^ Scientific Equipment).

Seeds were sown in polystyrene trays of 128 cells each, filled with Topstrato substrate (Vida Verde, BRA). Plants at the three to four true leaf stage were transplanted into 15-L pots (one plant per pot) containing a mixture of soil, sand, and composted cow manure (3:1:1). The soil texture was classified as sandy clay (sand, 52.9%; silt, 4.5%; clay, 42.6%). Fertilization and fungicide/insecticide applications were based on crop recommendations. The plants were tied to bamboo stakes placed inside each pot to prevent falling.

Each experiment was arranged in a randomized block design, with three replicates. Each replicate consisted of three plants arranged side-by-side (S1 Fig).

### Irrigation management and calculation

Tomato plants are more sensitive to drought during flowering and fruit set, [25, 26], therefore, this was the stage when drought conditions were imposed on the plants in environmental condition 2.

Under environmental condition 1, the soil water content was maintained at 100% available soil water (ASW) during the entire growing season. Under environmental condition 2, after the flowering stage, plants were subjected to drought stress by withholding water until the soil matric potential approached the wilting point ~ 0% ASW (~ −1500 kPa).

Irrigation management and calculations followed the methodology described by Dariva [27]. A soil sample was taken to estimate the soil water retention curve parameters based on the Van Genuchten equation [28] using SWRC Fit software [29]. To estimate the water tension value corresponding to 100% ASW, we assumed that field capacity was reached at a soil matric potential of −33 kPa [30]. To estimate when to stop water deprivation under environmental condition 2, we assumed that the wilting point would be reached at a soil matric potential of −1500 kPa [30]. Therefore, the plants were re-watered when the soil matric potential was estimated to be approximately −1490 kPa. After the drought stress period, the plants under both environmental conditions were kept well-watered until the end of the growing season.

Before filling them with soil, all pots were weighed to determine their weight (W_pot_). In addition, the bamboo stakes used in each pot were weighed to determine stake weight (W_stake_). Subsequently, all pots were filled with the same dry soil weight (W_ds_). The water weight (W_water_) of the first irrigation was determined by multiplying W_ds_ by the soil water content (kg/kg) at a soil water potential of −33 kPa.

The soil water content was monitored daily by weighing each pot. For the daily irrigations, the applied W_water_ was determined as total pot weight (W_totalpot_) minus the pot weight measured on that day. The total pot weight (W_totalpot_) was calculated as follows:

Wtotalpot=Wpot+Wds+Wwater+Wplant+Wstake (1)

where Wpot is the pot weight, Wds is the dry soil weight, Wwater is the water weight, Wplant is the plant weight, and Wstake is the bamboo stake weight. Wplant was determined by weighing same-aged spare plants grown within the experiment. One spare plant was harvested and weighed every 10 d to adjust Wplant value.

### Phenotyping

#### Physiological and morphological traits

Leaf water potential (LWP) was measured predawn (3:00–5:00) at the end of the stress period using a pressure chamber (model 3000; Soil Moisture, Santa Bárbara, CA, USA), according to the method described by [31]. LWP measurements were performed on young fully expanded leaves, the terminal leaflet and the one right bellow it, were used to take the measurements. The LWP value for each repetition was the mean value of two leaflets.

The leaf relative water content (LWC) was determined at the end of the drought stress period for three leaf samples taken from the top most fully expanded leaves. LWC was calculated according to [32] using the following formula:

$$LWC=\frac{LFW\;-\;LDW}{LSW\;-\;LDW}$$ (2)

where LFW is the fresh weight, LDW is the dry weight obtained after oven-drying for 48 h at 80°C, and LSW is the saturation weight determined after 24 h of re-saturation in tap water.

Shoot dry matter (SDM) was determined for the whole shoot (leaves and stems) at the end of the experiment by oven-drying for 48 h at 80°C. Shoot water content (SWC) was determined for the whole shoot (leaves and stems) at the end of the experiment using the following formula [32]:

$$Shoot\;water\;content=\frac{SFW\;-\;SDW}{SSW\;-\;SDW}$$ (3)

where SFW is the fresh weight, SDW is the dry weight obtained after oven-drying for 48 h at 80°C and SSW is the saturation weight determined after 24 h of re-saturation in tap water.

Fruit water content (FWC) was determined for a sample of three to four fully ripened fruits per plant picked randomly at the end of the experiment, according to the following formula [32]:

$$FWC=\frac{FFW\;-\;FDW}{FSW\;-\;FDW}$$ (4)

where FFW is the fresh weight, FDW is the dry weight obtained after oven-drying for 48 h at 80°C and FSW is the saturation weight determined after 24 h of re-saturation in tap water.

#### Yield

Tomato fruits were harvested when fully ripe during the course of the experiment. Fruits were considered fully ripe when they achieved stage red, meaning that more than 90% of the tomato surface, in the aggregate, was red. The fresh weight of all fruits from each plant was measured. The yield parameters consisted of total plant yield (Kg plant^−1^), mean fruit weight (MFW, g), and number of fruits per plant (N_fruits).

#### Fruit size attributes

The visual attributes—fruit length (FL) and width (FW)—of all harvested fruits were measured using a digital caliper (Louisware^®^ Stainless Steel Caliper 150mm/0-6 inch). Each fruit was visually evaluated to assess the presence of blossom-end rot (BER), scars, and cracking, and the results were expressed in terms of the percentage of fruits with defects.

#### Fruit quality attributes

Organoleptic quality measurements were performed randomly on four fully ripened fruits per plant. All four fruits per plant were homogeneous in size and color and were harvested on the same day. Fruit quality assessment began immediately after harvest.

Fruit firmness, described as the mean maximum penetration force required for pericarp rupture and expressed in Newtons (N), was measured using a digital penetrometer (model PDF-200, Soilcontrol, USA) fitted with a cylindrical stainless-steel probe with a round tip (Ø 8 mm). Two measurements, located 180º apart from one another, were taken in the equatorial region of each fruit.

After firmness measurement, the four selected fruits were macerated together in a blender to produce tomato juice, which was used to determine the total acidity (pH), total soluble solids (TSS), and titratable acidity (TA). The pH values of the juice samples were determined immediately using a benchtop pH meter (model pH 21, Hanna Instruments, Italy). TSS, expressed as °Brix, was determined using a digital refractometer (model HI 96801, Hanna Instruments, Italy). For TA measurements, samples of approximately 5 g of tomato juice were transferred to 100-mL volumetric flasks, which were then filled to capacity with distilled water. An aliquot of 10 mL of this solution was then titrated with a NaOH solution (0.005 mol L^−1^) using 1% phenolphthalein as an indicator. TA values were expressed as % citric acid and were obtained using the following formula:

$$TA=\frac{\left[A\times \;B\times \;C\times \;D\times \;100\right]}{grams\;of\;juice\;sample}$$ (5)

Where: A = (*mL NaOH*); B = (0.005 *N NaOH*); C = (0.064 *mEq acid citric factor*); D = (*correction* − *factor*).

Finally, the TSS/TA ratio was calculated as an indicator of flavor, as described by [33].

### Statistical analyses

A mixed-model methodology was adopted for statistical analyses via REML/BLUP (restricted residual maximum likelihood/best linear unbiased prediction) [34, 35], using R software and the Sommer package.

The statistical model was denoted by:

$$y=X\tau +Z_{e}\mu_{e}+Z_{g}\mu_{g}+Z_{i}\mu_{i}+e$$ (6)

where Y is the phenotypic data vector; *τ* is the fixed effects vector (overall mean and blocks); *μ*_*g*_ is the genotype effects vector (random), with $\mu_{g}\sim N\left(0,\sigma_{g}^{2}\right)$; *μ*_*e*_ is the environment effects vector (random), with $\mu_{e}\sim N\left(0,\sigma_{e}^{2}\right)$, *μ*_*i*_ is the genotype × experiment interaction (random), with $\mu_{i}\sim N\left(0,\sigma_{i}^{2}\right)$; e is the residual effects vector (random), with *e* ~ *N*(0,*R*); and X, Z_g_, Z_e_ and Z_i_ are incidence matrices for *τ*, *μ*_*g*_, *μ*_*e*_, and *μ*_*i*_, respectively. R is a diagonal matrix with residual variances, that is, $R=\overset{\underset{t}{i=1}}{⊗}\sigma_{e_{i}}^{2}$ where $\sigma_{e_{i}}^{2}$ is the residual variance in experiment i and t is the number of environmental conditions (in this case = 2).

For the random effects of the model, the significance of the likelihood ratio test was assessed using the chi-square statistic with one degree of freedom. Genetic values (BLUP means) were predicted for each of the 11 genotypes based on the 37 evaluated traits and two environmental conditions. For traits where significant genotype effects were detected, the BLUP means were used to estimate Pearson’s linear correlation coefficients.

#### Single-trait and multi-trait IL ranking

The genetic values (BLUP means) of traits that showed significant genotype effects (P < 0.05) were submitted to single-trait ranking, according to the designed ideotype: maximum values for FWC, N_Fruit, MFW, and yield; minimum values for BER; and mean values for TA. For each trait, the percentage difference of each IL was calculated relative to the commercial cultivar M-82. Subsequently, a multi-trait index based on factor analysis and genotype–ideotype design, known as the FAI-BLUP index, was used to rank the genotypes. Principal component analysis, factor analysis, ideotype determination, and genotype–ideotype distance calculation were performed in R software using the FAI-BLUP index routine developed by Rocha [36].

Principal component analysis was used to extract the genetic correlation matrix factorial loads obtained by the genetic values. The varimax criterion [37] was used for analytic rotation, and the weighted least squares method [38] was used to calculate the factor scores. The number of ideotypes was defined based on a combination of desirable and undesirable factors for the selection objective. The following algorithm provides the number of ideotypes:

$$NI=2^{n}$$ (7)

in which, *NI* is the number of ideotypes and n is the number of factors.

After ideotype determination, genotype–ideotype distances were estimated and converted into spatial probabilities, enabling genotype ranking. The following algorithm was used.

$$P_{ij}=\frac{\frac{1}{d_{ij}}}{\sum_{i=1;j=1}^{i=n;j=m}\frac{1}{d_{ij}}}$$ (8)

where P_ij_ is the probability that the i^th^ genotype (i = 1, 2,…, n) is similar to the j^th^ ideotype (j = 1, 2,…, m) and d_ij_ is the genotype–ideotype distance from the i^th^ genotype to the j^th^ ideotype, based on the standardized mean Euclidean distance.

### Candidate gene identification

The genomic segments of *S*. *pennellii* introgressed into the ILs selected by the FAI-BLUP index were further analyzed to identify candidate genes for drought tolerance, adapting the methodology described previously [39]. The tomato IL population map provided in [40] was utilized, which delineates individual chromosomes with restriction sites for introgressed segments and all identified marker genes.

To automatically identify all marker genes from each chosen IL in the HTML code, we modified the script created by Toubiana [39]. We then utilized information on the corresponding orthologs in the *Arabidopsis thaliana* genome [41] to infer the functionality of each gene that was identified. The identification of candidate genes for drought tolerance was based on the relevance of their functionality to this process.

## Results

### Analysis of deviance

**Fig 1:**
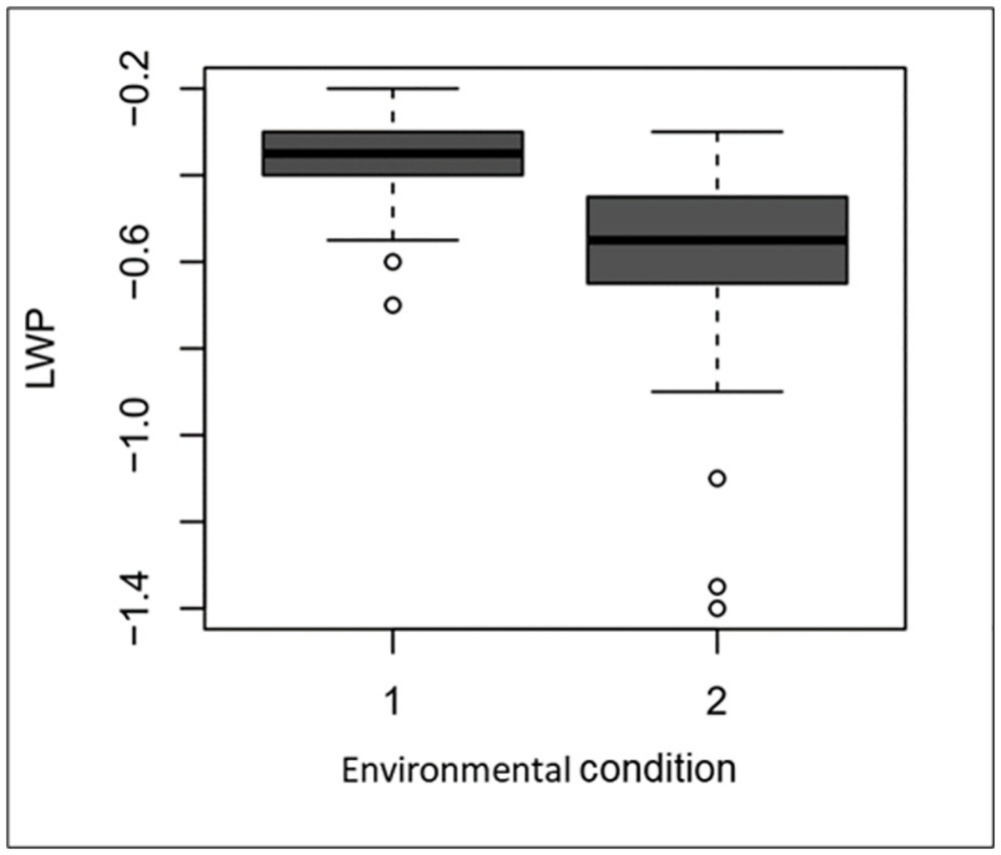
Boxplot of tomato ILs leaf water potential (LWP) in the two environmental conditions. Environmental condition 1 = non-stressed plants, environmental condition 2 = plants subjected to drought stress. Circles indicate outliers in the dataset.

The effect of environment was significant for LWP (p-value > 0.05), meaning that the mean LWP of the two environmental conditions were different. Fig 1 shows a boxplot of LWP under environmental conditions 1 and 2. As expected, the values observed for environmental condition 1, in which the plants were well-watered throughout the growing cycle, were higher than those observed for environmental condition 2, in which the plants were subjected to drought stress by water deprivation. Even the outliers observed in environmental conditions 1 and 2 follow a similar pattern, in environmental condition 1 genotypes displayed higher LWP than in environmental condition 2.

Among the 18 evaluated traits, significant genotypic differences were found for five yield and fruit quality attributes: FWC, MFW, FL, FW, BER, and TA. The effect of the interaction between genotype and environmental conditions was not significant for any of the evaluated traits.

### Pearson’s correlation

**Fig 2:**
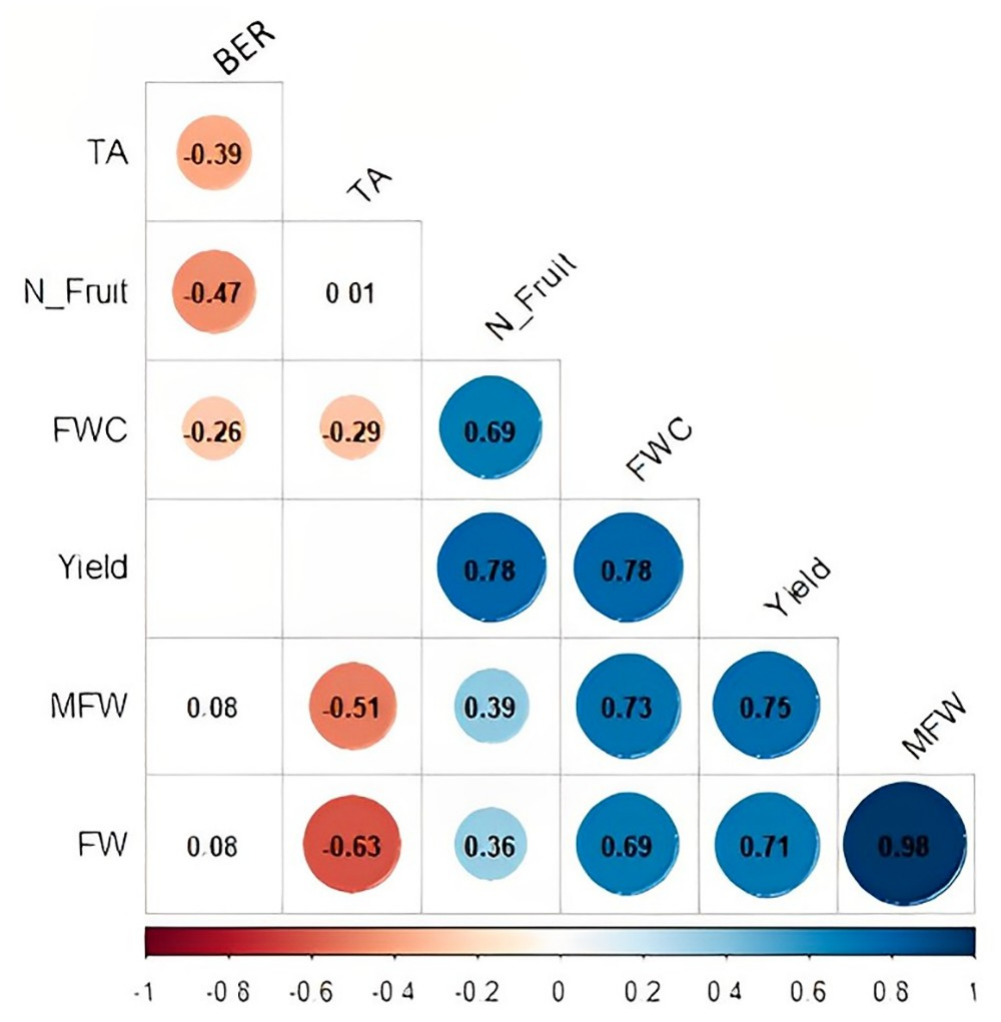
Pearson’s correlation matrix of BLUP means for all traits showing significant genotypic effects. Correlations were validated by the t-test at 0.05 significance level, and blank squares mean that the correlation was not significant (p > 0.05). BER = blossom-end rot, FW = fruit width, FWC = fruit water content, MFW = mean fruit weight, N_fruits = mean number of fruits per plant, TA = titratable acidity, Yield = total yield. Colors are related to the correlation values. The darker the red the closer to -1 and the daker the blue the closer to 1.

Our study on the associations between the yield and fruit quality attributes revealed 19 significant linear correlations, of which 13 were positive and six negative (p < 0.05, t-test) (Fig 2). Overall, the yield attributes were positively correlated. As MFW displayed a strong positive correlation with FW (r = 0.98), we decided to retain only one of them for further analysis and removed FW. The other correlations among the traits were only moderate (r < 0.7); hence, all the other traits were retained. Yield showed a moderate positive correlation with N_ fruit (r = 0.78), FWC (r = 0.78), and MFW (r = 0.75).

### Single-trait and multi-trait ranking of ILs subjected to drought stress

**Fig 3:**
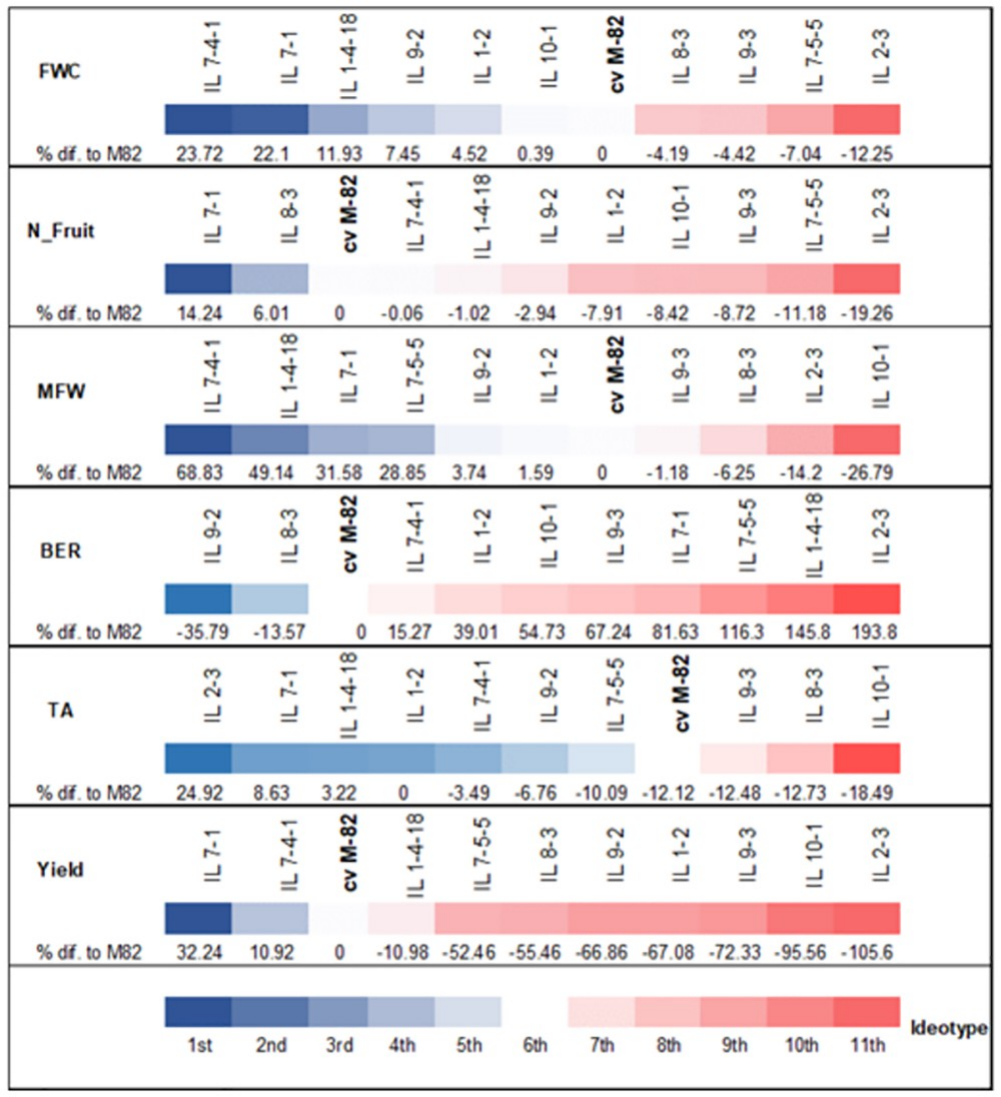
Ranking of genotypes according to the ideotype for each trait and the percentage difference (increase or decrease) in relation to cv. M-82. The genotypes closest to the ideotype are on the left. The colored gradient indicates the intensity of difference in relation to M-82: the deeper the blue and red colors, the more substantial the changes promoted by the S. pennnellii genome away from the ideotype. BER = blossom-end rot, FWC = fruit water content, MFW = mean fruit weight, N_fruits = mean number of fruits per plant, TA = titratable acidity, Yield = total yield.

Fig 3 displays the genotype ranking according to the ideotype for each trait. The ideotype was designed considering maximum values for FWC, N_Fruit, MFW, and yield, minimum values for BER, and mean values for TA. The chromosomal fragments of *S*. *pennellii* introgressed into cv. M-82 promoted changes in all the ILs for all traits. IL7-1 stood out in terms of yield-related traits. Compared with its parent, cv. M-82, there was an impressive increase in mean fruit weight (68.83%) and total yield (32.24%). This IL also had the highest increase in fruit number. IL 7-4-1 was among the top five genotypes for all traits, and the only trait that was not improved by the *S*. *pennellii* introgression was BER. When analyzing all the traits together, it is evident that an introgressed region can increase some traits while decreasing others. In addition, a genotype ranked first for one trait could be ranked last for others. For example, the introgression of *S*. *pennellii* promoted an increase of 49.14% in the MFW of IL 1-4-18; this IL was also ranked second for FW and penultimate for BER.

**Fig 4:**
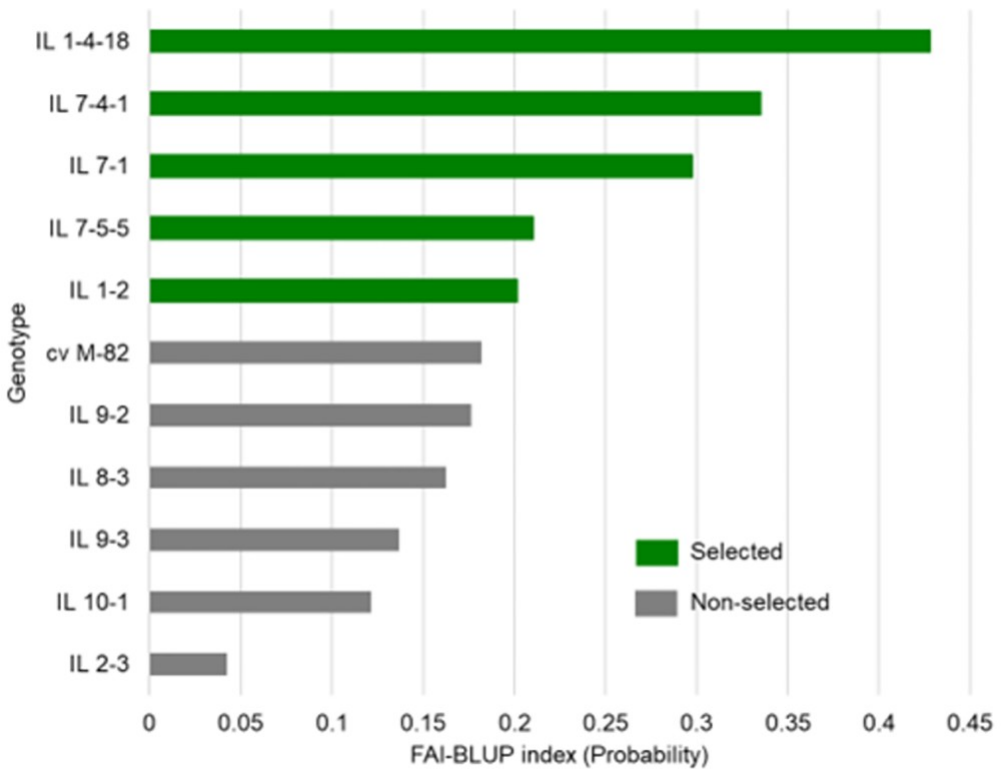
Genotype ranking and selected genotypes using the FAI-BLUP index.

Fig 4 presents the ranking of the 11 evaluated genotypes according to the multi-trait index FAI-BLUP, and the probability of good drought tolerance in relation to the distance to the ideotype. This method ranked the genotypes according to the proposed ideotype (maximum values for FWC, N_Fruit, MFW, and yield; minimum values for BER; and mean values for TA), considering all the parameters simultaneously. The cultivar M-82 was ranked the sixth closest to the drought-tolerant ideotype. Therefore, the five ILs ranked above it were selected as drought-tolerant and investigated for the presence of candidate genes for this trait. Among the five ILs selected as drought-tolerant, IL 1-4-18 and IL-1-2 were previously determined to be drought-tolerant in the germination and seedling stages. In addition, the most drought-tolerant plants at the germination and seedling stages (IL 1-4-18) were also the most drought-tolerant during the vegetative and reproductive stages.

### Candidate genes associated with drought tolerance

**Fig 5:**
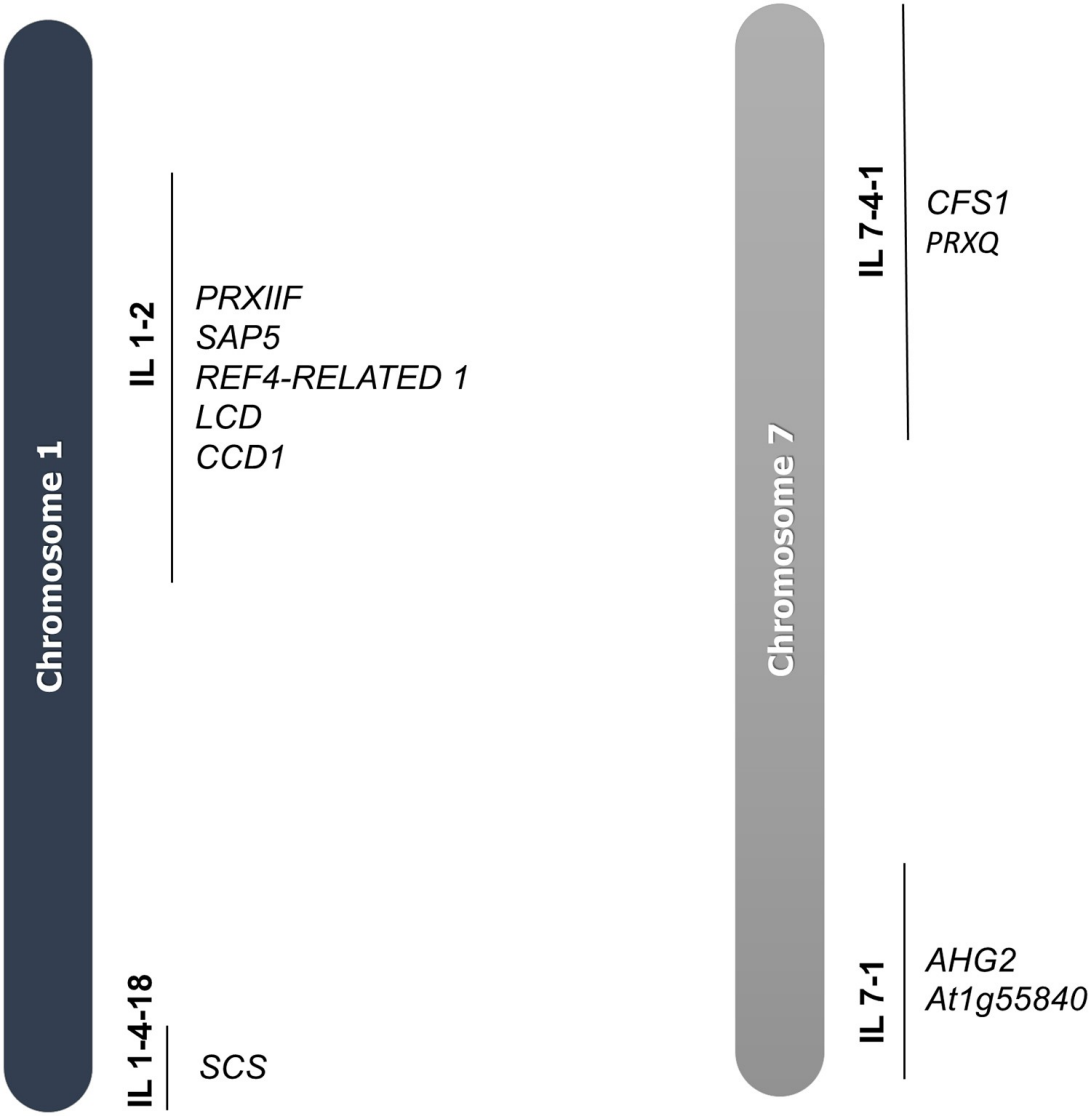
Candidate genes associated with drought tolerance in the selected ILs.

All the genotypes ranked above M-82 (IL 1-4-18, IL 7-4-1, IL 7–1, IL 7-5-5, and IL 1–2) were further investigated for candidate genes associated with drought tolerance. Gene markers associated with the introgressed segments of the selected ILs were identified using [40]. Their functionality was inferred using information on their respective orthologs in the *A*. *thaliana* genome. Fig 5 provides a summary of all the candidate genes discovered.

In IL 1-4-18, the gene *SCS* was identified. It encodes SnRK2, a protein involved in plant response to water deprivation [42]. In IL 7-4-1, *PRXQ* and *CFS1* were identified. *PRXQ* encodes peroxiredoxin Q, which decomposes peroxides and is therefore involved in the cellular response to oxidative stress [43]. *CFS1* encodes a RING-type zinc-finger family protein involved in the response to ABA [44].

In IL 7–1, the genes *At1g55840* and *AHG2* were identified. *At1g55840* encodes a Sec14p-like phosphatidylinositol transfer family protein that is involved in the defense response to abiotic stress [45]. *AHG2* encodes a poly(A)-specific ribonuclease AtPARN. The expression of AtPARN is upregulated by ABA or stress treatment, acting upstream of or within the response to ABA and osmotic stress [46]. No genes related to drought tolerance were found in the selected IL 7-5-5.

In IL 1–2, the genes *PRXIIF*, *SAP5*, *REF4-RELATED 1*, *LCD*, and *CCD1* were identified as candidates. *PRXIIF* encodes a mitochondrial matrix localized peroxiredoxin involved in redox homeostasis, which is involved in oxidative stress response [47]. *SAP5* encodes a protein with E3 ligase activity that positively regulates stress responses in Arabidopsis and responds to water deprivation [48]. *REF4-RELATED 1*, encodes a protein shown to physically associate with the conserved transcriptional coregulatory complex, mediator, and is involved in the regulation of phenylpropanoid homeostasis. It regulates the phenylpropanoid metabolic process related to plant response to abiotic stress [49]. *LCD* encodes an enzyme that decomposes L-cysteine into pyruvate, H2S, and NH3, which is involved in plant response to drought [50]. Finally, *CCD1* encodes 9-cis-epoxycarotenoid dioxygenase, which responds to water deprivation [51].

## Discussion

An ideal drought-tolerant genotype must display drought tolerance at all developmental stages of plant growth [12]. Therefore, specific stages throughout the ontogeny of the plant must be evaluated separately [13]. Here, we selected genotypes previously screened and ranked for drought tolerance during seed germination and seedling growth and evaluated them during the vegetative and reproductive stages under two water regimes (100% ASW and drought exposure imposed by water withholding for up to 20 d).

The significant difference among the environments observed for LWP (p < 0,05) highlights that the adopted methodology was efficient in promoting drought stress in the plants from which water was withheld. Even the outliers displayed a much lower LWP when submitted to drought stress. Despite the wide range of methodologies proposed to induce drought stress in tomatoes [13, 27, 52, 53], multiple studies, from the pioneering work of Wudiri [25] to the recent experiments of Cui [26], suggest that the flowering and fruit-setting stage is when tomato plants are the most sensitive to drought. Therefore, promoting severe water deprivation (soil almost reaches wilting point) during this stage, as we did in our study, proved to be an accurate method of screening and selecting plants exhibiting drought tolerance.

As expected, the plants from environmental condition 2, which were exposed to drought, showed lower LWP than those from environmental condition 1. Predawn LWP usually corresponds to a daily maximum or base water potential, which is presumed to correspond to the equilibrium between the soil and plant water potentials [54]. It is believed that predawn plant water potential is in equilibrium with the “wettest” soil accessed by the roots [55, 56]. Therefore, the predawn LWP was used as a surrogate for the soil water potential. After irrigation suspension in environmental condition 2, evapotranspiration consumed the water available in the soil over the entire period of drought stress. By the end of the period, the soil reached a much lower water potential than that in the pots that were watered daily.

We did not identify significant genotype × environment relationships for any of the evaluated traits. Like most herbaceous plants, 90% of the total fresh weight of tomato plants is water [57]. For tomato fruits, this percentage is even higher; water accounts for 93–95% of tomato composition [15]. All chemical and biochemical cellular processes, including photosynthesis, take place within an aqueous media. Water deficit immediately stops cell division and enlargement and stem and leaf elongation, processes that require a minimum turgor pressure. A lack of water also inhibits photosynthesis, closes stomata, and decreases respiration rates and other enzyme-mediated processes [58]. Thus, it was expected that the genotypes under drought stress would show different responses for the evaluated traits than those under watered conditions. The LWP values confirmed that the plants in environmental condition 2 were under drought stress conditions. However, the drought period was short, considering the extent of the growth cycle, which was approximately five months. A hypothesis for the non-interaction between genotype and environment is that after drought stress suspension, the plants subjected to water deficit adjusted their physiological and biochemical machinery to adapt to these conditions. This suggests that they might be a valuable set of genetic resources for the further investigation of drought tolerance.

Genotypic differences (p < 0,05) were identified for only seven of the 18 evaluated traits. This can be explained by the fact that the 11 genotypes used as plant material were highly genetically related (10 ILs from the collection developed by [20] and their genitor, the commercial cultivar M-82). Therefore, it is likely that the genomic portions shared among the genotypes, rather than the introgressed fragments, are associated with the traits for which significant genotypic differences were not observed. In this study, the differences promoted by the introgressed fragments are the ones of interest; therefore, only the traits displaying genotypic differences were further analyzed.

The chromosomal fragments of *S*. *pennellii* introgressed into cv. M-82 promoted changes in fruit quality- and yield-related traits (FWC, MFW, FL, FW, yield, BER, and TA) of ILs. M-82 was not the first-ranking genotype for any of these, indicating that the genomic introgressions were favorable for some of the ILs. While *S*. *pennellii* produces small green fruits unlike cultivated varieties, certain wild alleles may exhibit counterintuitive effects compared to what one would expect when examining the phenotype of a crop’s wild relative [12]. While examining the function of wild tomato alleles in a background of tomato cultivars through QTL analysis, were identified two ILs with larger fruit sizes than the cultivated parent, and QTL mapping in the IL population revealed that the allele leading to larger fruit size was, surprisingly, contributed by *S*. *pennellii* [20]. Moreover, pyramiding different genetic loci derived from *S*. *pennellii* into *S*. *lycopersicum* lines in a heterozygote state increased fruit yield by 30–50% under field conditions compared to an elite variety [59].

From the single-trait ranking, it is evident that an introgressed region can increase some traits while decreasing others. In addition, a genotype ranked first for one trait could be ranked last for others. Therefore, single-trait ranking is not helpful in selecting the genotypes best suited to drought conditions during the vegetative and reproductive stages, justifying the use of a multi-trait approach.

The FAI-BLUP index ranked five ILs above M-82, confirming that the *S*. *pennellii* genomic fragments in these ILs increased their drought tolerance. *S*. *pennellii* possesses several adaptive mechanisms that ensure its survival in arid environments, including morphophysiological and anatomical modifications in the aerial part of the plant, such as a cuticular composition associated with increased resistance to water flux, a smaller leaf surface area, and greater leaf thickness. Therefore, it was expected that the introgressed *S*. *pennellii* fragments in the ILs would carry genes that enhance drought tolerance. Moreover, IL 1-4-18 and IL-1-2, which were ranked as drought-tolerant in this study, were also ranked as drought-tolerant in their germination and seedling stages. Drought tolerance is a stage-specific phenomenon that is controlled by many genes with different effects [14, 15, 32]. Therefore, most probably, the genes controlling the drought tolerance of IL 1-4-18 and IL-1-2 during germination and seedling growth were not the same as those controlling this trait in the stages evaluated in our study. Hence, the fragments of *S*. *pennellii* inherited by these ILs are a rich source of drought tolerance-related genes, with genes acting during seed germination and seedling growth, and genes expressed during the vegetative and reproductive stages. Our results suggest that IL 1-4-18 and IL-1-2 are promising genotypes for inclusion as genitors in breeding programs aimed at developing drought-tolerant tomato cultivars.

IL 1-4-18 was ranked the most drought-tolerant genotype and the gene *SCS*, which encodes SnRK2, was identified in this IL. SnRK2 was described as a crucial regulator of plant responses to abiotic stresses [60]. This protein acts as a positive central regulator of ABA signaling during water stress at the vegetative and reproductive stages [42]. Water deficit stress, such as drought, triggers various biochemical and physiological responses in plants, including alterations in gene expression and the accumulation of the phytohormone ABA [61]. ABA regulates diverse plant processes, including the adaptation of plants to water stress [62, 63]. Numerous drought stress-responsive genes have been reported, many of which are induced by ABA [64]. SnRK2s pathways regulate the plant response to ABA by the direct phosphorylation of various downstream targets, such as SLAC1, KAT1, AtRbohF, and transcription factors required to express numerous stress response genes [60]. Therefore, the gene discovered in our study might have played a central role in the response of IL 1-4-18 to water deprivation, resulting in drought tolerance.

In IL 7-4-1, the genes *PRXQ* and *CFS1* were identified. *CFS1* encodes a RING/FYVE/PHD-type protein. This group of proteins is nuclear ubiquitin E3 ligases. Although they do not seem to be directly involved in transcriptional regulation, they can interact with some transcription factors to regulate their transcriptional activities, resulting in improved abiotic and biotic stress tolerance in several species [44]. *PRXQ* encodes peroxiredoxin Q, which plays a role in the cellular response to oxidative stress caused by peroxide decomposition. Peroxiredoxins are part of the enzymatic ROS-scavenging system developed by plants to scavenge ROS generated during biotic stress events such as drought [65]. Peroxiredoxin Q, one of the four plant subtypes, is a chloroplast gene associated with responses to several abiotic stresses [43]. By scavenging peroxides generated under stress, peroxiredoxin Q protects the photosynthetic machinery, enabling photosynthesis to continue in non-optimal conditions. IL 7-4-1 was ranked as the second-most drought-tolerant genotype and showed outstanding behavior for all evaluated traits under drought conditions. *PRXQ* and *CFS1* may be key genes in the drought-tolerance strategy of this genotype.

In IL 7–1, the genes *At1g55840* and *AHG2* were identified. *At1g55840* encodes a Sec14p-like phosphatidylinositol transfer family protein. In soybeans, this protein is a component of a stress response pathway that serves to protect adult plants under osmotic stress [66]. Proteins with a Sec14p-like domain play a role in the lipid regulation of the Rho-mediated signaling pathway, which is involved in water stress tolerance in wild barley [67]. *AHG2* encodes AtPARN. Although the specific roles of this protein are not well-described, they were found to be expressed when plants were subjected to drought stress [46], and according to Covarrubias [68] they are involved in the post-transcriptional gene regulation of salinity and drought responses. These two genes are good candidates for explaining the drought tolerance of IL 7–1.

Although IL 7-5-5 was determined to be the fourth closest genotype to the drought tolerance ideotype (Fig 3), we were unable to identify any candidate genes that could account for this finding. The adopted methodology excluded genes that lacked an *A*. *thaliana* ortholog or had unknown functions from the list of potential candidate genes. Hence, it is possible that one or several of these excluded genes are related to the drought tolerance displayed by IL 7-5-5 in this study.

In IL 1–2, the genes *PRXIIF*, *SAP5*, *REF4-RELATED 1*, *At3g61140*, *LCD*, *At3g62010*, *CCD1*, *At4g15530*, and *At5g49480* were identified as potential candidates. *PRXIIF* encodes a mitochondrial matrix localized peroxiredoxin involved in redox homeostasis, which is involved in oxidative stress response. Under less than optimal conditions, such as drought and salinity, this mitochondrial thioredoxin is necessary for the effective operation of key metabolic pathways, including antioxidant metabolism and stomatal function [47]. Upon experiencing drought episodes, Arabidopsis plants become more drought-resistant as a result of metabolite adaptations in both primary and secondary metabolism triggered by the deactivation of the mitochondrial TRX system [69]. As this is a mitochondrial trait, IL 1–2 should be considered a maternal donor for use in breeding programs. *SAP5* encodes a protein with E3 ligase activity that acts as a positive regulator of stress responses. In wheat (*Triticum aestivum*) and *Arabidopsis thaliana*, STRESS-SAP5 is involved in drought tolerance and acts as an E3 ubiquitin ligase to target DRIP and MBP-1 for degradation [70]. *REF4-RELATED 1* is involved in the regulation of the phenylpropanoid metabolic process [71]. The phenylpropanoid biosynthetic pathway is activated under abiotic stress conditions such as drought, resulting in the accumulation of various phenolic compounds, which, among other roles, scavenge harmful ROS [49]. It was found that an elevated phenylpropanoid content enhanced wheat tolerance to drought stress by maintaining an improved water status, leading to higher photosynthesis rates and lower membrane damage [72]. The gene *LCD* encodes L-cysteine desulfhydrase. L-cysteine desulfhydrase has been identified as the primary catalyst for the breakdown of cysteine into H2S, which is critical in the regulation of stomatal closure in plant responses to drought stress [73]. H2S may serve as a vital intermediary in the ABA-mediated regulation of ion channels involved in stomatal control. H2S influences the expression of ABA receptor candidates, and ABA, in turn, affects H2S production. As a result, H2S interacts with ABA to regulate the stomatal activity, thereby contributing to drought stress tolerance in Arabidopsis [50]. In addition, rice plants exhibit enhanced drought tolerance when LCD is overexpressed, leading to increased H2S production and the persulfidation of total soluble protein [6]. *CCD1* encodes 9-cis-epoxycarotenoid dioxygenase, which is involved in the response to water deprivation. This enzyme is thought to be a key enzyme in ABA biosynthesis [74] involved in stress responses, and is quickly accumulated by many plant species when exposed to drought stress [63]. 9-cis-epoxycarotenoid dioxygenase and the response to water deprivation was linked by demonstrating that drought induces the expression of the gene encoding this enzyme, which in turn controls the level of endogenous ABA under drought-stressed conditions [51].

Uncovering genes in IL populations is a powerful method for mining genetic variation present in wild crop relatives for traits with the potential to improve desired characteristics in elite varieties. Several traits identified in ILs and advanced background populations have been used in tomato breeding [21]. In this study, we identified several candidate genes related to drought tolerance in ILs. We provide useful information for classical breeding approaches by indicating promising genotypes to be used as parents, and for molecular breeding approaches by identifying candidate genes that can be introgressed into elite varieties through marker-assisted selection. Our results, therefore, represent a significant step toward developing drought-tolerant tomato cultivars.

## Conclusion

The FAI-BLUP index ranked IL 1-4-18, IL 7-4-1, IL 7–1, IL 7-5-5, and IL 1–2 as closest to the drought-tolerant ideotype. The introgressed lines IL 1-4-18 and IL1-2 were also previously identified as drought-tolerant during the germination and seedling stages. The following candidate genes associated with drought tolerance were identified in selected ILs: *AHG2*, *At1g55840*, *PRXIIF*, *SAP5*, *REF4-RELATED 1*, *PRXQ*, *CFS1*, *LCD*, *CCD1*, and *SCS*. Because they are already associated with genetic markers, they can be transferred to elite tomato cultivars through marker-assisted technology after validation.

## Supporting information

S1 Fig Diagram illustrating the trial set. Environmental condition 1 = plants were kept in non-stressed conditions throughout the growing season, with the soil water content maintained at field capacity. Environmental condition 2 = plants were challenged with drought by withholding water for up to 20 d after flowering. 1 to 11 indicates the genotypes, 1 = IL 1-4-18, 2 = IL 2–3, 3 = IL 1–2, 4 = IL 9–2, 5 = IL 10–1, 6 = IL 8–3, 7 = IL 7-4-1, 8 = IL 7-5-5, 9 = IL 9–3, 10 = IL 7–1, 11 = cv. M-82.
(TIF)

S1 File Data set underlying data used to reach the conclusions drawn in the manuscript. (XLSX)
