# Supplementary figures and images for: Uncovering tomato candidate genes associated with drought tolerance using Solanum pennellii introgression lines
Source: PLoS One. 2023 Jun 15;18(6):e0287178. doi: 10.1371/journal.pone.0287178 (PMC10270355; doi:10.1371/journal.pone.0287178)

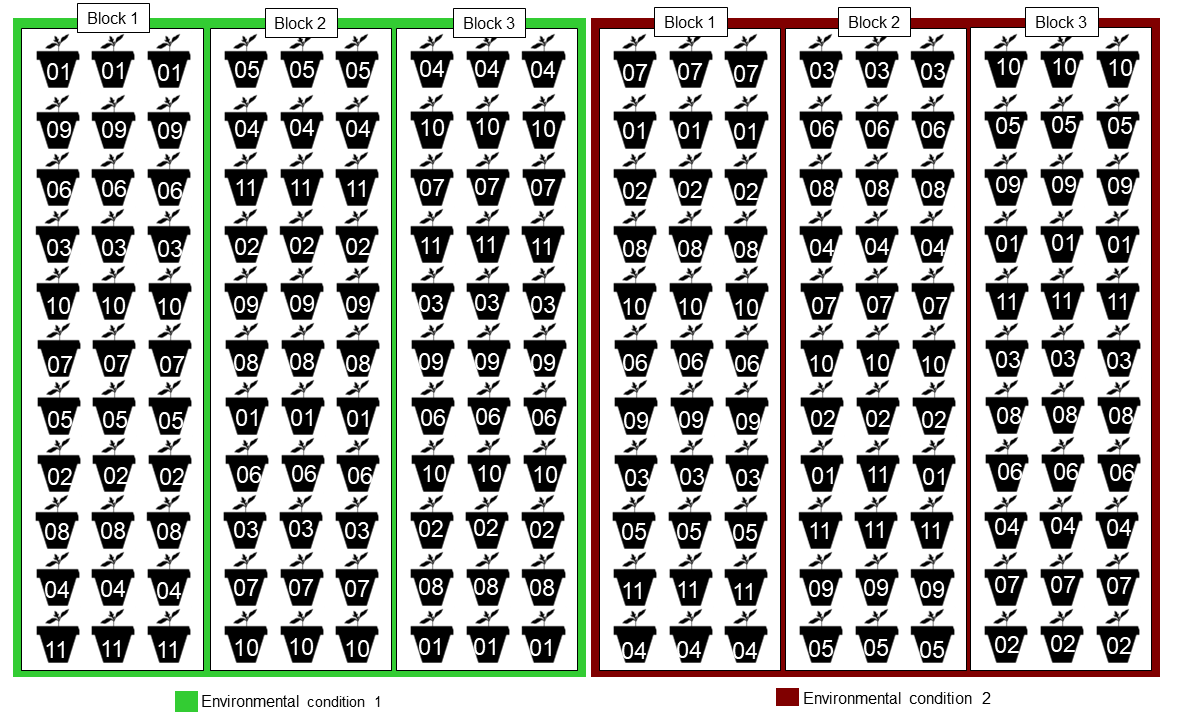

Supplement: S1 Fig — Environmental condition 1 = plants were kept in non-stressed conditions throughout the growing season, with the soil water content maintained at field capacity. Environmental condition 2 = plants were challenged with drought by withholding water for up to 20 d after flowering. 1 to 11 indicates the genotypes, 1 = IL 1-4-18, 2 = IL 2–3, 3 = IL 1–2, 4 = IL 9–2, 5 = IL 10–1, 6 = IL 8–3, 7 = IL 7-4-1, 8 = IL 7-5-5, 9 = IL 9–3, 10 = IL 7–1, 11 = cv. M-82. (TIF) [file pone.0287178.s001.tif]
